# Nonspecific cervical spine pain/neck pain/in medical personnel of north-eastern Poland—A cross-sectional study

**DOI:** 10.3389/fmed.2024.1466370

**Published:** 2024-12-09

**Authors:** Anna Citko, Stanisław Górski, Ludmiła Marcinowicz, Cybulski Mateusz, Sierakowska Matylda

**Affiliations:** ^1^Akademia Medyczna Nauk Stosowanych i Holistycznych, Warsaw, Mazowieckie Voivodeship, Poland; ^2^European University of Applied Medical and Social Sciences, Olsztyn, Warminsko-Mazurskie Voivodeship, Poland; ^3^Department of Medical Education, Jagiellonian University Medical College, Kraków, Malopolskie Voivodeship, Poland; ^4^Department of Obstetrics, Gynecology and Maternity Care, Faculty of Health Sciences, Medical University of Bialystok, Bialystok, Podlaskie Voivodeship, Poland; ^5^Department of Integrated Medical Care, Faculty of Health Sciences, Medical University of Bialystok, Bialystok, Podlaskie Voivodeship, Poland

**Keywords:** nurses, paramedics, neck pain (NP), spondylogenic pain syndrome, predictive factors

## Abstract

Both mechanical and psychological overload are inherent to the work of nurses and paramedics, resulting spondylogenic pain.

**Aim of the study:**

To identify potential risk factors influencing the prevalence of non-specific cervical spine pain in professionally active nurses and paramedics.

**Material and methods:**

324 nurses (53.2% of the total) and 285 paramedics (46.8%) were included in the study−609 people in total. The study was carried out using an auditorium survey technique supervised by the researchers. The methods used were: Nordic Musculoskeletal Questionnaire, a spinal pain questionnaire validated according to IEA guidelines, a short version of the IPAQ and the author's survey questionnaire, concerning sociodemographic data, chronic diseases including metabolic syndrome (MetS). A univariate logistic regression model was used in the statistical analysis. The level of statistical significance was taken as *p* < 0.05.

**Results:**

Recurrent NP was significantly more common in paramedics compared to nurses (29.5 % vs. 9.3 %; *p* < 0.0001). In a univariate logistic regression model, the risk of NP was significantly increased by: length of service > 15 years (*p* < 0.024), presence of: low back pain (*p* < 0.0001), type 2 diabetes (*p* = 0.013), hypertension (*p* < 0.001), depression (*p* < 0.01). Of the modifiable factors, the risk of NP was significantly increased by high physical activity and short sleep <7 h (*p* < 0.001).

## Introduction

Musculoskeletal pain, especially the pain of spinal origin, known as spondylogenic pain is a considerable health concern in our times (1). In most cases, its cause cannot be determined, which is why it may be defined as nonspecific pain. Only in about 10% of cases can the cause of pain be identified (specific pain) (2).

Neck pain (NP) is among the most common types of musculoskeletal pain. (3, 4). It belongs to ailments with a rather poorly recognized pathophysiology (5). Its causes include a non-ergonomic posture during work, negative emotions, stress, and lifting heavy weights. (6–8). It is emphasized that neck pain is characteristic of jobs requiring bending and straightening of the neck as well as jobs involving spinal rotation (9). The increase in nonspecific neck pain may also result from the widespread use of electronic devices, which results in a forced body position when using a computer, mobile phone, or tablet (10). Observations show that the loss of cervical lordosis, which is one of the first radiological signs of the increased tension in the paraspinal muscles, occurs due to frequent chin-to-chest bending. It turns out that in the case of bending at an angle of 60 degrees, there is an overload of the cervical spine, reaching even 25–26 kg, while in the case of a neutral position, that overload is 4–5 kg (2, 11).

Both mechanical and psychological overload is an inherent part of the work of nurses and paramedics. Working in direct contact with patients often requires lifting (parts of the body or the entire patient), remaining in one forced body position for a long time (most often in forward flexion), and frequent bending. Moreover, the work related to saving lives, especially for paramedics, additionally induces stress and results in increased muscle tension and spondylogenic pain (8, 12). It may be presumed that a recurrent neck pain in the medical personnel may not only worsen their quality of life but also result in a decrease in the effectiveness of care they provide (13).

Given the importance of the health concern at issue, the question arises as to which risk factors, related solely to the professional work of nurses and paramedics, play a significant role in the occurrence of a recurrent neck pain.

A significant portion of the published studies analyses the impact of various factors on the occurrence of spondylogenic pain, with particular classification into: individual factors (age, gender, body mass, sedentary lifestyle), morphological factors, and psychosocial factors. The opinions of the authors regarding the relationship of those factors with nonspecific spondylogenic pain are divided (2, 7, 14). However, only few reports describe the impact of chronic diseases on the occurrence of neck pain. Studies suggesting their association with risk factors for cardiovascular diseases are interesting (15–17).

It should also be emphasized that only very few epidemiological studies in Poland address the frequency of occurrence of neck pain in the professional group of paramedics in Poland (18). It seems reasonable, therefore, to conduct a multi-aspect analysis that may help identify 'new' predictive factors and implement better preventive strategies, both for nurses and paramedics.

The aim of the study has been to identify potential risk factors influencing the frequency of nonspecific neck pain occurrence in actively working nurses and paramedics in northeastern Poland.

## Materials and methods

### Materials

The research was conducted from October 2016 to June 2017. The study is a continuation to deepen the analysis of the database created in 2017 to the extent of the research on the occurrence of spondylogenic pain in nurses and paramedics. The established database served as the source of variables used in previous publications (14, 17).

The sample size needed to participate in the study was calculated using the IBM SPSS Statistics engine-based PS IMAGO Pro programme. It was estimated based on an indication of the test power value and the effect size strength of interest. The test power value was taken as 80 %. With regard to the strength of the effect size, an educated guess was used, namely the effect size in the population was estimated on the basis of available sources. The value was 0.5, with a default error of 5% for type I and 20 % for type II. The significance level was *p* < 0.05.

Purposive sampling was used to recruit the group for the cross-sectional study. The fact that the study was carried out in three voivodship hospitals in the north-eastern Poland region and in ambulance stations was in favor of maintaining the fixed power level of the test (equal to 80 %) and not exceeding this limit. All people available at that time and place who met the inclusion criteria were therefore recruited. Our sample is therefore the largest possible. Questionnaires were given directly to the respondents.

The study involved nurses and paramedics working in northeastern Poland. A total of 324 nurses (53.2% of the total nursing workforce) and 285 paramedics (46.8%) participated in the study, totalling 609 individuals. The study group was divided by age: 302 individuals aged 30–40 years (49.6%) and 307 individuals aged 41–60 years (50.4%). In the study group, there were 247 men (40.6%) and 362 women (59%). Among the medical personnel surveyed, the female gender predominates. This is due to the fact that the profession of a nurse is mainly carried out by women in Poland. Therefore, we did not obtain an even distribution of respondents in terms of gender.

Due to gender, in the group of nurses 324 individuals (100%) were women and in the paramedic group there were 38 (13.3%) women and 247 (86.7%) men. For the purpose of the further statistical analysis related to the topic of this paper, a group of 114 individuals complaining of neck pain was selected (54 paramedics, 60 nurses).

### Methods

The study was conducted by means of the diagnostic survey method utilizing the following research tools:

- The questionnaire was based on the standardized Nordic Musculoskeletal Questionnaire containing questions assessing the frequency of the musculoskeletal pain occurrence in 9 anatomical regions: neck, shoulders, upper back, lower back, elbows, wrists/hands, hips/thighs, knees, ankles/feet. Respondents were also tasked with marking on a diagram the location of any discomfort if present. An episode of pain was considered “recurrent” if it occurred ≥3 times within a year, and its total duration did not exceed 12 weeks (19). The NMQ is repeatable, sensitive (sensitivity 0.90 for cervical spondylosis) and useful as a screening and surveillance tool (20)
- The questionnaire of low back pain and other spine sections, validated according to the guidelines of the International Epidemiological Association (IEA), containing questions regarding the occurrence of spine pain, coexisting symptoms, lifestyle, chronic diseases, and injuries to specific spine sections within the 3 months prior to completing the questionnaire (21).
- The proprietary questionnaire regarding socio-demographic data, profession, workplace for nurses, selected lifestyle elements (smoking, coffee consumption, self-assessment of health status), in-depth interviews regarding chronic diseases as well as coping strategies for spondylogenic pain in the year preceding the completion of the questionnaire.
- The short version of the International Physical Activity Questionnaire (IPAQ) containing questions about the frequency and duration of undertaken physical activity of high, moderate, and low intensity, lasting continuously for at least 10 min (22, 23). The percentage share of participants considered sufficiently active was determined based on the estimated caloric expenditure of physical activity, following the assumptions of Paffenbarger et al.

The results were classified according to the following criteria:

- Insufficient physical activity (<600 MET·min/week); sufficient physical activity (between 600 and 1,500 MET·min/week);
- Elevated physical activity (1,500–3,000 MET·min/week, but <3 days a week of intense efforts);
- High physical activity (above 1,500 MET·min/week but at least 3 days a week with intense efforts, or at least 3,000 MET·min/week) (23)

Studies in 14 countries (12 scientific centers) demonstrated the usefulness, validity and reliability of the questionnaire (22).

Taking into account the specifics of the workplace and its association with spine load, nurses were divided into three groups:

- Group I—Working in hospital emergency departments or ambulance services,
- Group II—In other “heavy-duty” departments (surgical units, neurology, intensive care units (ICU), oncology, geriatrics),
- Group III—In “light-duty” departments or outpatient clinics (14, 17)

Respondents treated for autoimmune diseases, cancer, inflammatory spinal disease, individuals with a history of osteoporotic fracture, as well as those who had experienced injuries within the 3 months preceding the questionnaire completion, respondents who had undergone spinal surgery (including discectomy), and those with congenital cervical spine anomalies (for example, respondents with Kimmerle's anomaly) were excluded from the study.

A sleep duration of <7 h per day was considered insufficient, and coffee consumption of ≥6 cups of coffee per day was considered excessive (14, 17, 24, 25).

Within the framework of the assessment of the impact of diseases on the occurrence of a recurrent nonspecific neck pain, the following conditions were taken into account: components of metabolic syndrome: hyperlipidemia, hypertension, type 2 diabetes, depression, and/or anxiety disorders.

The Body Mass Index (BMI) was calculated based on the given height and weight values.

The detailed distribution of age, seniority, and anthropometric parameters in the study group are provided in Table 1.

**Table 1:** Detailed distribution of age, seniority, and anthropometric parameters in the study group.

**Variable**	**Median**	**Minimum**	**Maximum**	**Q_1_**	**Q_3_**	p^*^
Age (years)	41.0	30.0	55.0	35.0	47.0	<0.1
Seniority (years)	20	1.0	35.0	15	26	<0.01
Height (cm)	170	150	201	164	178	<0.001
Weight (kg)	72	46	110	64	82	<0.001
BMI (kg/m ^2^)	24.8	17.96	38.46	22.34	27.66	<0.01

^*^Due to the non-normal distribution of the variables presented in the Table, the Median, Minimum, Maximum Values for Q1, and Q3 have been provided (p < 0.05; Shapiro-Wilk Test).

The occurrence of cervical spondylosis was determined based on the description of the cervical spine X-ray provided by the respondent. The examination was conducted through individual meetings between the researcher and the respondent at the healthcare facility.

The influence of potential predictive factors was assessed depending on the occupational group, taking into account individual risk factors, including those related to lifestyle, job characteristics, as well as the co-occurrence of musculoskeletal disorders and chronic civilization-related diseases. The analysis of the frequency of occurrence of hypothetical predictive factors for a recurrent back pain is presented in Table 2.

**Table 2:** Distribution of hypothetical predictive factors among group of paramedics, group of nurses and total study group.

**Predictive factor**		**Group of paramedics; *n* (%)**	**Group of nurses *n* (%)**	**Total study group *n* (%)**
**Individual risk factors**				
Age	30–40 years	160 (56.14)	140 (43.21)	300 (49,26)
	41–60 years	125(43.86)	184 (56.79)	309 (50,74)
Overweight or obesity		143 (50.18)	153 (47.22)	296 (48.6)
Smoking		191(67.02)	179 (55.25)	370 (60.76)
Excessive coffee consumption (≥6 cups of coffee daily)		31 (10.88)	67 (20.68)	98 (16.09)
Unhealthy dietary behavior		196(68.77)	175 (54.01)	371 (60.92)
Sedentary lifestyle		131 (45.96)	171 (52.78)	302 (49.59)
High physical activity		88 (30.88)	56 (17.28)	144 (23.65)
Insufficient hours of sleep (<7 h/day)		149 (52.28)	162 (50)	311 (51.07)
Residence in rural area		39 (13.68)	46 (14.2)	85 (13.96)
Family history of lower back pain		83 (29.12)	62 (19.14)	145 (23.81)
Family history of neck pain		19 (6.67)	17 (5.25)	36 (5.91)
Low self-rated health status		31 (10.88)	53 (16.36)	84 (13.79)
**Occupational risk factors**				
Seniority	>15 years	187 (65.61)	243 (75)	433 (71,1)
	≤15 years	98 (34.39)	78 (24.07)	176 (28,9)
Shift work		285 (100)	291 (89.81)	576 (94.58)
Working as a paramedic		285 (100)	0 (0)	285 (46.8)
**Musculoskeletal symptoms and conditions**				
Recurrent cervical spine pain		54 (18.95)	60 (18.52)	114 (18.72)
Recurrent thoracic spine pain		73 (25.61)	37 (11.42)	110 (18.06)
Cervical spondylosis		30 (10.53)	18 (5.56)	48 (7.88)
Scoliosis		43 (15.09)	65 (20.05)	108 (17.73)
Peripheral joint osteoarthritis		71 (24.91)	73 (22.53)	144(23.65)
Recurrent lower back pain		134 (47.02)	119 (36.37)	253 (41.54)

During the research, a database was established as a source of variables, containing the respondents' statements, opinions, and evaluations, allowing for the application of computational techniques. The statistical analysis began with a substantive and logical examination of the collected data. In the first stage, the conformity of the continuous variables with a Gaussian distribution and their distribution were verified. In all of the cases, the Shapiro-Wilk test was conducted, and the hypothesis of normal distribution was rejected. The comparison of the significance of two or more proportions of nominal variables was performed by means of the chi-square independence test. To dichotomous variables, the logistic regression analysis was applied (the odds ratio was calculated).

The odds ratio (OR) for two comparable groups, Group A (exposed to the factor) and Group B (not exposed to the factor), is defined as the ratio of the “odds” of occurrence in Group A to the “odds” of occurrence in Group B, i.e., OR.

The odds ratio (OR) was calculated according to the following formula:


$$\begin{array}{c} OR_{\text{A}/\text{B}}=\frac{S\left(A\right)}{S\left(B\right)}=\;\frac{\frac{P\left(A\right)}{1-P\left(A\right)}}{\frac{P\left(B\right)}{1-P\left(B\right)}}=\;\frac{P\left(A\right)\cdot \left(1-P\left(B\right)\right)}{P\left(B\right)\cdot \left(1-P\left(A\;\right)\right)} \end{array}$$

Where:

OR, odds ratio

Interpretation of the Odds Ratio:

OR = 1 indicates equivalence of the odds in the compared groups

OR > 1 indicates that the chance of the event occurring (e.g., developing the condition) in Group A is greater than in Group B

OR < 1 indicates that the chance of the event occurring in Group A is less than in Group B

The studies have been approved by the Bioethical Commission of the Medical University of Białystok (R-I-002/261/2016).

## Results

**Figure 1:**
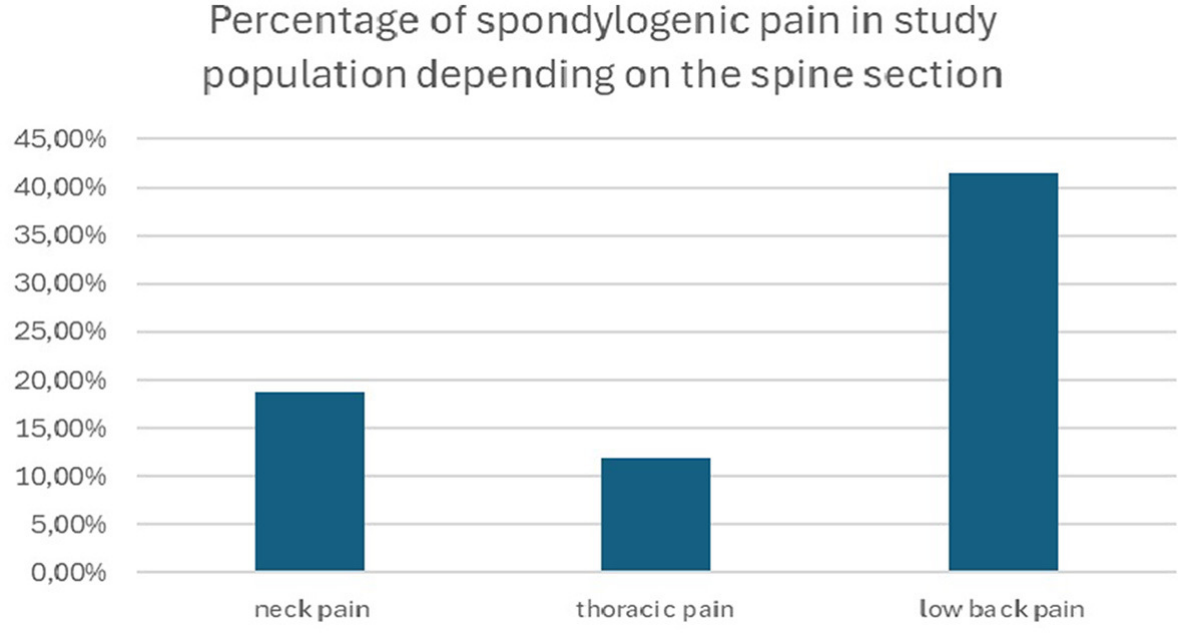
Percentage of spondylogenic pain in the study population depending on the spine section.

A nonspecific recurrent neck pain was reported by 114 (18.7%) of the respondents (from the total group of 609 individuals). As a matter of comparison, 253 (41.5%) respondents complained of low back pain, and 73 (11.9%) of them reported pain in the thoracic region (Figure 1).

Paramedics reported a recurrent neck pain significantly more often as compared to nurses (*p* < 0.0001) (Table 3).

**Table 3:** Assessment of the frequency of a recurrent neck pain (C) based on the profession (A, B).

**Study group**	**Recurrent neck pain (C)**		**df**	**Chi-square**	***p***
	**Yes**; ***n*****(%)**	**No;** ***n*** **(%)**			
Paramedics (A)	84 (29.7%)	201 (70.5%)	1	40.72	<0,0001
Nurses (B)	30 (9.3 %)	294 (90.7%)			

df, Degree of freedom.

Chi-square, Test statistical value.

p, Statistical significance.

It is important to note that in the group of nurses, only 31 (9.6%) of them reported working in hospital emergency departments or emergency medical services, while 151 (46.6%) of them worked in departments classified as “heavy,” and 142 (43.8%)—in departments classified as “light” or outpatient clinics.

Nurses working in hospital emergency departments reported a recurrent neck pain significantly more often as compared to nurses working in “heavy” and “light” departments (*p* < 0.0001) (Table 4).

**Table 4:** Association between workplace (A, B, C) and recurrent neck pain (D).

**Nurses' workplace**	**Recurrent neck pain (D)**		**df**	**Chi-square**	***p***
	**Yes**; ***n*** **(%)**	**No**; ***n*** **(%)**			
“Heavy-duty” departments (A)	10 (6.6)	141 (93.4)	2	86.53	<0.0001
“Light-duty” departments (B)	3 (2.1)	139 (97.9)			
Emergency departments, emergency medical services (C)	17 (54.8)	14 (45.2)			

df, Degree of freedom.

Chi-square, Test statistical value.

p, Statistical significance.

The analyses have shown that the risk of a recurrent neck pain was significantly higher in the presence of individual predictive factors associated with a high physical activity (*p* < 0.001), insufficient hours of sleep (<7 h/day) (*p* < 0.001), a history of neck pain in the respondent's family (*p* < 0.001), and low self-rated health (*p* < 0.001).

The risk of neck pain was significantly higher in the case of the profession of a paramedic (*p* < 0.001) and amongst the personnel with longer years of seniority (>15 years) (*p* = 0.024).

Statistically significant risk factors for neck pain also include discomfort and conditions from other segments of the spine presented in Table 5.

**Table 5:** Impact of hypothetical predictive factors on recurrent neck pain among group of subject.

**Predictive factor**		**Respondents with recurrent neck pain; *n* (%)**	**OR**	**95% confidence interval for OR**	***p***
**Individual risk factors**					
Age [years]	41–60 years old	63 (21.0)	1.345	0.893–2.025	0,156
	30–40 years old	51 (16.5)	—	—	—
Overweight or obesity		56 (18,9)	1.026	0.683–1.542	0.902
Smoking		73 (19,7)	1.187	0.778–1.811	0.427
Excessive coffee consumption (≥6 cups of coffee daily)		17 (17,4)	0.896	0.508–1.580	0.704
Unhealthy dietary behaviour		71 (19,1)	1.073	0.706–1.633	0.741
Sedentary lifestyle		59 (19,5)	1.113	0.740–1.672	0.608
High physical activity		44 (30,5)	2.483^*^	1.605–3.841	<0.001
Insufficient hours of sleep (<7 hours/day)		75 (24,1)	2.111^*^	1.380–3.229	<0.001
Residence in rural area		9 (10,6)	0.473^**^	0.229–0.974	0.042
Family history of neck pain		29 (80,6)	23.785^*^	10.096–56.035	<0.001
Low self-rated health status		27 (32,1)	2.385^*^	1.429–3.981	<0.001
**Occupational risk factors**					
Seniority [years]	>15 years	91 (21.0)	1.770^*^	1.078–2.905	0,024
	≤15 years	23 (13.1)	—	—	—
Shift work		108 (18,7)	1.039	0.418–2.577	0.935
Working as a paramedic		84 (29,5)	4.1^*^	2.601-6.448	<0.0001
**Musculoskeletal symptoms and conditions**					
Recurrent thoracic spine pain		50 (68.5)	16.033^*^	9.171-28.028	<0.0001
Cervical spondylosis		38 (79.2)	24.250^*^	11.600–50.693	<0.001
Scoliosis		42 (38.9)	3.792^*^	2.393–6.009	<0.001
Postural defects excluding scoliosis		20 (22.7)	1.336	0.774–2.307	0.299
Peripheral joint osteoarthritis		70 (48.6)	5.382^*^	3.573–8.107	<0.001
Recurrent lower back pain		76 (30.0%)	3.593 ^*^	2.336-5.527	<0.0001
**Extravertebral conditions**					
Hyperlipidemia		12 (26.7)	1.647	0.822–3.299	0.159
Hypertension		15 (44.1)	3.796^*^	1.865–7.727	<0.001
Type 2 diabetes		18 (31.0)	2.133^*^	1.173–3.880	0.013
Depression and/or anxiety disorders		11 (39.4)	3.003^*^	1.366–6.602	0.006

^*^The chances of a recurrent neck pain occurrence are significantly increased.

^**^The chances of a recurrent neck pain occurrence are significantly decreased.

OR, Odds Rate.

Among extraspinal disorders predisposing to a recurrent neck pain, arterial hypertension (*p* < 0.001) as well as depression and/or anxiety disorders (*p* = 0.006) and type 2 diabetes (*p* = 0.013) may be listed (Table 5).

The studies have also indicated that the odds of a recurrent neck pain are significantly reduced in individuals residing in rural areas (*p* = 0.05)

Taking into account the coping strategies for neck pain, 102 respondents (89% of those with a recurrent neck pain) took painkillers. However, only 44 individuals (39%) sought advice from a doctor and/or physiotherapist. Only 38 respondents (33.3%) reported to have participated in training on ergonomic principles.

## Discussion

Many reports highlight the growing problem of nonspecific neck pain (NP). The data analysis from the Global Burden of Disease has shown an increase in the prevalence and incidence of neck pain worldwide, from ~276.5 million cases in 1990 to around 475.2 million cases in 2019 (26). The annual prevalence rate is 37.2% (range 16.7–75.1%) (27). Neck pain, immediately after lower back pain, is considered to be the most common, complex musculoskeletal pain condition with multifactorial influence, encompassing pain, disability, and reduced quality of life (28–31).

Healthcare workers have developed various measures for managing and preventing neck pain. However, paradoxically, neck pain is very common among them, likely due to the nature of their work (29).

The results of our own research showed the presence of nonspecific/recurrent neck pain in almost 1/5 of the actively working medical personnel (18.7%). Meanwhile, recurrent pain in the lumbar spine affected nearly a half of the respondents participating in our study.

Puszczałowska-Lizis et al., arrived at slightly different conclusions. They included 50 nurses and 50 paramedics, aged 40–50 years old, who had experienced neck and/or lower back pain incidents in the last 2 years. The researchers found that neck and lower back pain occurred with equal frequency (19).

Habibi et al., conducted a survey study with a group of 247 nurses from Iran, aged 23–67 years old. They found that 27.5% reported to have experienced neck pain 1–2 times per week, while 9.3% reported to have experienced it daily (8). However, it should be emphasized that the percentage share of neck pain (NP), according to some authors, is significantly higher. In the study conducted by Yang et al., involving a group of 2,170 nurses employed in 12 hospitals in China, as many as 61.8% reported to have experienced NP in the year preceding the study (32).

The lower percentage share of neck pain observed in our study, as compared to the studies cited above, may be due to the fact that we took into account the individuals with a recurrent neck pain. We did not take into account the individuals who had experienced any single incident of neck pain within the last 12 months.

Many researchers have demonstrated a clear connection between musculoskeletal disorders and occupational load. There is no doubt that a significant portion of the tasks performed by nurses involves physical labor. The most common causes leading to neck pain have been proven to primarily include muscle and ligament overloads, resulting from poor posture, weak work ergonomics, and consequently, excessive muscle fatigue. Exposure to repetitive arm or neck and shoulder movements, which is a common occurrence in the work of both nurses and paramedics, has been found to be among the physical factors related to occupational tasks that contribute to the development of neck pain (6, 7, 33).

Our research also confirms the impact of working conditions in the group of paramedics, among whom NP occurred significantly more frequently as compared to nurses (29.6% vs. 9.3%; *p* < 0.001). It is likely related to a higher number of emergency cases involving awkward neck movements. It bears noting that Bryndal et al., obtained a similar percentage share of NP among the surveyed Polish paramedics (36% out of the 201 surveyed) (34).

On the other hand, the work of nurses varies in terms of responsibilities and tasks performed. Nurses working in various hospital departments have different duties as compared to those employed in outpatient clinics. The results of our research confirm the association of a recurrent NP with work-related factors (35). NP occurred significantly more frequently among nurses working in hospital emergency departments as compared to those working in “heavy” and “light” departments (6.62% vs. 2.11%; *p* < 0.001).

Some researchers suggest that a preceding NP incident is the strongest predictor of NP recurrence. Therefore, the first incident should be a warning sign of the potential development of early degenerative changes in the cervical spine (6, 11, 24).

Although the diagnosis of cervical spondylosis in our study was based on the description of a radiological image, which may have raised some concerns, the results have suggested a connection with work-related strain as well as certain genetic predispositions. Firstly, paramedics significantly more frequently reported the occurrence of cervical spondylosis as compared to nurses (10.53% vs. 5.56%; *p* < 0.05). Secondly, the presence of NP in the respondent's family increased the risk of NP in the surveyed individuals by almost 24 times (*p* < 0.001).

Although we did not find any correlation between the age of the respondents and the occurrence of a recurrent NP, a correlation with the length of seniority was found. In the univariate logistic regression model, medical workers with more than 15 years of seniority had almost twice the risk of experiencing NP (*p* < 0.005). It may suggest that the physical demands associated with professional work had a greater impact on the occurrence of spondylogenic pain than the degenerative process of the spine associated with age.

The impact of physical activity on neck and lower back pain remains controversial. Our previous studies have shown that a sedentary lifestyle increases the risk of developing spondylogenic pain (14). However, there are many reports indicating that excessive physical activity also adversely affects the function of the musculoskeletal system (36).

According to the results of our study, a high physical activity (including physical exertion related to occupational work, household chores, commuting, and recreation) more than doubles the likelihood of experiencing nonspecific NP (*p* < 0.001). It may be related to the fact that fatigued muscles have a reduced ability to protect the spinal tissues and the adaptive tissue remodeling process that opposes micro-injuries (28, 31).

The search for new predictors of NP, including among diseases classified as civilization-related, is important. Undoubtedly, identifying factors related to the development of neck pain will promote the development of preventive strategies and early interventions aimed at improving the quality of life and preventing disability.

In the related literature, there are occasional reports of coexistence of neck pain in patients with metabolic syndrome (MetS). Some researchers suggest that components of metabolic syndrome, such as hyperlipidemia or type 2 diabetes may contribute to premature disc degeneration resulting from insufficient supply of nutrients to the disc cells (15, 16). Mäntyselkä et al., studying a group of 135 individuals, found neck pain in 7.9% of men without metabolic syndrome (MetS) and 16% among those with MetS. The respective proportions among women were 16% and 25% (37).

In our own study, among the medical personnel we observed a twofold higher occurrence of neck pain in patients with type 2 diabetes (*p* = 0.013), while in patients with hyperlipidemia, the presence of neck pain was more frequent but without any statistical significance. Similar conclusions were reached by Lima Florencio et al., who examined 2095 individuals with diabetes and 2,095 healthy individuals. Neck pain was significantly more common in individuals with diabetes (15). On the other hand, in the study conducted by Ahorukomeye et al., among 304,627 residents of the United States, neck pain was significantly more common in individuals with hyperlipidemia (16).

On the other hand, hypertension among nurses, in the study conducted by Iizuka et al., was not a predictive factor for neck pain (32). However, in our own study, we found nearly a fourfold higher occurrence of neck pain among those treated for hypertension (*p* < 0.001).

In the related literature, the coexistence of neck pain with depression or anxiety is also emphasized. It has been observed that patients with neck pain exhibit greater emotional disturbances than the compared control group of healthy individuals (2).

Taking into account the professional group of nurses and paramedics, occupational factors related to the risk of developing nonspecific neck pain include the elements of task-related and social stress, such as fast-paced work and stress. They are listed as psychosocial risk factors for nonspecific neck pain (38). Furthermore, due to the economic situation faced by medical personnel in Poland, working multiple jobs or staying “after hours” can exacerbate fatigue and stress, potentially leading to nonspecific neck pain symptoms.

Researchers emphasize that poor mental state (tension, stress, lack of support, anxiety) adversely affects the condition of the cervical spine. Psychological tension may lead to static overload of tendons, muscles, ligaments, and joints. Gravity-defying muscles, that are essential for maintaining proper posture, are particularly susceptible to the said overload (8, 32).

Our own research findings confirm all that, showing that depression and/or anxiety disorders triple the risk of a recurrent spondylogenic neck pain (*p* < 0.01).

As far as other modifiable risk factors for neck pain are considered, their occurrence is significantly influenced by low self-rated health and insufficient sleep (<7 h), both of which are found to more than double the likelihood of neck pain. That is supported by the research conducted by Chin et al. (39).

Our study results also point to a concerning trend regarding pain management. It's associated with frequent use of painkillers and less frequent utilization of medical advice or physiotherapy among medical professionals. It might reflect a broader issue because, on the one hand, it poses risks of medication-related complications, and on the other hand, improper treatment may lead to disability (40). It's important to emphasize that the study group comprises healthcare workers who should be aware of the consequences of such disorders. Additionally, it's concerning that only 33% of the respondents reported to have participated in ergonomics training (41).

In summary, our own research confirms the significant impact of selected psychosocial and ergonomic factors on the occurrence of a recurrent nonspecific NP. The studies also suggest that comorbidities, especially the components of metabolic syndrome, may be considered significant “new” predictors of NP. However, that requires further research. It is important to emphasize the limitations of the studies, that were questionnaire-based. The variables were estimated based on a self-report, so some measurement error was inevitable. Additionally, cervical spondylosis was diagnosed based on descriptions of examination results rather than one's own evaluation of radiographs or MRI scans.

Therefore, in order to confirm significant correlations, additional studies covering more rigorous research designs and measurements are needed. Such studies should incorporate objective methods for assessing work practices and include elements of clinical examination.

### Clinical implications

The conducted studies indicate that recurrent non-specific neck pain is a significant health problem in both nurses and paramedics. Therefore, there is a need for continuous monitoring of this issue and the implementation of preventive programs within these groups.

Regarding prevention, the results suggest the need to educate healthcare staff about work ergonomics, promote a healthy and balanced lifestyle, and ensure proper and professional therapeutic management.

When determining treatment methods for patients with recurrent neck pain, the knowledge of NP risk factors, including comorbidities like those related to metabolic syndrome (MetS), plays a crucial role. Failing to consider these factors may result in ineffective physiotherapy and kinesitherapy.

Cuenca-Zaldivar et al. demonstrated the beneficial effects of a therapeutic exercise program, including self-mobilization exercises for the neck and lumbar regions, on pain intensity (38). The literature also highlights the effectiveness of manual therapy in patients with chronic neck pain. Fernández-Carnero et al., in their studies, not only attempted to identify the key predictive factors for recovery in patients with chronic neck pain but also to determine the treatment method depending on the subtype of neck pain. By using the Mulligan mobilization technique in the studied group, they showed that the greater the anxiety and the lower the range of lateral flexion, the higher the probability of successful treatment with this technique (3). Therefore, it may be worth considering the inclusion of these techniques in prevention and treatment programs for neck and back pain among medical personnel.

### Study limitations and future directions

The study was survey-based, with variables estimated through self-reporting, making some degree of measurement error unavoidable. Additionally, cervical spondylosis was diagnosed based on a description of the condition rather than an independent evaluation of radiographic or MRI images.

Therefore, to confirm significant relationships, further studies using more rigorous designs and measurements are needed. These should include objective methods of assessing work practices and incorporate elements of clinical examination. This is especially relevant for comorbidities, particularly cardiovascular risk factors.

An emerging challenge would be to study the impact of e-addictions and the Fear of Missing Out (FOMO) syndrome on the occurrence of recurrent non-specific neck pain. Such studies could use survey questionnaires like the FOMO Scale by Andrew K. Przybylski or the Bergen Facebook Addiction Scale (42).

Assessing patient expectations before and after treatment for chronic non-specific neck pain, as well as analyzing the prognostic value of these expectations, seems important.

## Conclusions

1. Neck pain occurs in nearly one-fifth of the surveyed medical staff, with higher frequency among paramedics and individuals with longer work experience (>15 years).

2. Selected psychosocial and ergonomic factors significantly influence the occurrence of non-specific recurrent NP. Individual predictors include high physical activity, sleep <7 h, family history of pain, and low self-assessed health.

3. Comorbidities, particularly components of metabolic syndrome (MetS) and depression, can be considered important “new” predictors of neck pain.

## Data Availability

The datasets presented in this study can be found in online repositories. The names of the repository/repositories and accession number(s) can be found in the article/supplementary material.
